# Provision of Lipid-Based Nutrient Supplements to Mothers During Pregnancy and 6 Months Postpartum and to Their Infants from 6 to 18 Months Promotes Infant Gut Microbiota Diversity at 18 Months of Age but Not Microbiota Maturation in a Rural Malawian Setting: Secondary Outcomes of a Randomized Trial

**DOI:** 10.1093/jn/nxz298

**Published:** 2020-01-07

**Authors:** Arox W Kamng'ona, Rebecca Young, Charles D Arnold, Noel Patson, Josh M Jorgensen, Emma Kortekangas, David Chaima, Chikondi Malamba, Ulla Ashorn, Yin B Cheung, Per Ashorn, Kenneth Maleta, Kathryn G Dewey

**Affiliations:** 1 Department of Biomedical Sciences, University of Malawi, College of Medicine, Blantyre, Malawi; 2 Institute for Global Nutrition and Department of Nutrition, University of California, Davis, Davis, CA 95616, USA; 3 School of Public Health and Family Medicine, University of Malawi, College of Medicine, Blantyre, Malawi; 4 Center for Child Health Research, University of Tampere Faculty of Medicine and Life Sciences, 33100 Tampere, Finland; 5 Program in Health Services & Systems Research and Center for Quantitative Medicine, Duke-NUS Medical School, Singapore 169857; 6 Department of Pediatrics, Tampere University Hospital, 33520 Tampere, Finland

**Keywords:** lipid-based nutrient supplements, multiple micronutrients, gut microbiota, infants diet, microbiota diversity

## Abstract

**Background:**

Diet may alter the configuration of gut microbiota, but the impact of prenatal and postnatal nutritional interventions on infant gut microbiota has not been investigated.

**Objective:**

We evaluated whether providing lipid-based nutrient supplements (LNSs) to mother–infant dyads promotes a more diverse and mature infant gut microbiota, compared to maternal supplementation with multiple micronutrients (MMN) or iron and folic acid (IFA).

**Methods:**

We enrolled 869 pregnant women in a randomized trial in Malawi. There were 3 study groups, with women receiving 1 MMN capsule daily during pregnancy and 6 mo postpartum, or 1 LNS sachet (20 g) daily during pregnancy and 6 mo postpartum, or 1 IFA capsule daily (during pregnancy) then a placebo daily (postpartum). Infants in the LNS group received LNS from 6 to 18 mo; infants in the other groups did not receive supplements. The infants’ fecal microbiota were characterized by PCR amplification and sequencing of the bacterial 16S rRNA gene (variable region 4). The primary outcomes were microbiota α diversity and maturation [as microbiota-for-age *z* score (MAZ)]. Specific associations of taxa with intervention were established with indicator species analysis (ISA).

**Results:**

Primary outcomes did not differ between IFA and MMN groups, so these groups were combined (IFA + MMN). Mean ± SD α diversity was higher in the LNS group at 18 mo for Shannon index [3.01 ± 0.57 (LNS) compared with 2.91 ± 0.60 (IFA + MMN), *P* = 0.032] and Pielou's evenness index [0.61 ± 0.08 (LNS) compared with 0.60 ± 0.09 (IFA + MMN), *P* = 0.043]; no significant differences were observed at 1, 6, 12, or 30 mo. MAZ and β diversity did not differ at any age. We found 10 and 3 operational taxonomic units (OTUs) positively associated with LNS and IFA + MMN, respectively; however, these associations became nonsignificant following false discovery rate correction at 10%.

**Conclusions:**

Prenatal and postnatal LNS intake promoted infant gut microbiota diversity at 18 mo, after 12 mo of child supplementation, but did not alter microbiota maturation. This trial was registered at clinicaltrials.gov as NCT01239693.

## Introduction

Malnutrition is responsible for about 45% of deaths in children globally (1), and is common in Sub-Saharan Africa (2). Among the nutritional interventions to prevent maternal and child undernutrition and micronutrient deficiencies (3), the provision of small-quantity lipid-based nutrient supplements (SQ-LNSs) to mothers during pregnancy and lactation, and to their infants starting at 6 mo after birth is a relatively new strategy (4). Two recent meta-analyses assessing the impact of SQ-LNS supplementation have shown positive effects on birth outcomes (5) and child growth (6), although there is heterogeneity among trials.

Intake of nutrients and other food substances may influence gut microbiota development, immunity, and metabolism in both animals (7, 8) and humans (9–13), both positively and negatively. For example, oligosaccharides present in human milk, and to a lesser extent in bovine milk, can strongly influence microbiota composition (14, 15). Gut microbes may also respond differentially to the amounts and types of fat and protein in the diet (16–18). Amounts of micronutrients may also affect the microbial community. In Kenya, infants recruited at age 6 mo and given nutritional supplements containing iron for 4 mo were shown to have an increased abundance of pathogenic species and increased cases of intestinal inflammation (19). By contrast, studies in Malawi revealed that iron-containing LNS or corn-soya blend given to children did not have an impact on the gut microbiota profile at age 18 mo (20, 21). A recent study showed that growth of pathogens such *Escherichia coli* and *Salmonella typhimurium* was significantly reduced when independently cultured in an iron-deficient in vitro colonic fermentation model (22). However, no reduction was observed in growth of beneficial bacteria species such as *Lactobacillus rhamnosus* (22).

The interventions in the 2 Malawian studies included LNS supplementation to children only, with no prenatal component. Maternal prenatal diet has recently been associated with infant gut microbiota, depending on delivery mode (23). However, the impact of LNS supplementation to both mothers and children on the diversity and maturity of the gut microbiota has not previously been explored.

This article reports secondary outcomes from a randomized controlled clinical trial, the iLiNS-DYAD-M trial, which was designed to study the health impacts of SQ-LNS when provided to Malawian mothers during pregnancy and the first 6 mo postpartum and to their newly born children from 6 to 18 mo of age (24). We have previously shown that provision of SQ-LNS did not increase the mean birth length or weight in the full study population, but that there may have been a modest impact among selected subgroups (25). There was also no significant impact on child growth at 18 mo (24). We report herein the effects of the intervention on the children's gut microbiota composition and maturation. We tested the hypothesis that SQ-LNS supplementation to both mothers and their infants would lead to a more diverse and mature gut microbiota between ages 1 and 30 mo, compared to maternal supplementation with multiple micronutrients (MMN) during pregnancy and the first 6 mo of lactation only or iron and folic acid (IFA) during pregnancy only. The hypothesis was based on the fact that LNS contained MMN plus 4 additional minerals, protein and fat and was administered to both mothers and their children, unlike IFA or MMN which were given to mothers only.

## Methods

### Study setting and design

**FIGURE 1:**
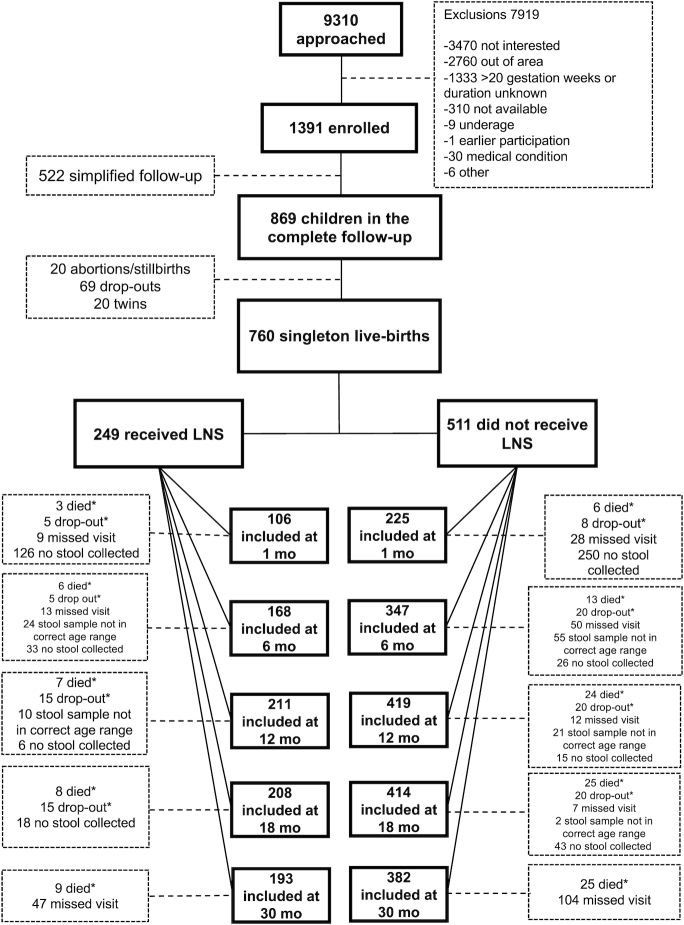
Flow diagram of recruitment, enrollment, and follow-up of Malawian women and their infants who participated in the International Lipid-Based Nutrient Supplements Project. *Death and drop out numbers are cumulative. LNS, lipid-based nutrient supplement.

This study was part of a randomized, controlled, partially blinded, parallel-group clinical trial known as the International Lipid-based Nutrient Supplements DYAD (iLiNS-DYAD) trial. Details of the study, including the original sample size calculation are described elsewhere (25). Briefly, the iLiNS-DYAD trial was conducted in 2 hospitals and 2 health centers in a rural area in Mangochi district, Malawi, a setting with high prevalence of infant stunting and underweight, and poor food security. The trial enrolled 1391 pregnant mothers aged ≥15 y and ≥20 weeks of gestation from the antenatal clinics of the 2 hospitals and 2 health centers (**Figure 1**). Women were excluded from the trial if they had chronic medical conditions, pregnancy complications at enrollment (moderate to severe edema, blood hemoglobin concentration <50 g/L, systolic blood pressure >160 mmHg or diastolic blood pressure >100 mmHg), were previously enrolled in iLiNS-DYAD, or concurrently enrolled in another clinical trial.

All 1391 women enrolled were advised to consume supplements until delivery, while a subset of 869 women were advised to continue with the supplementation until 6 mo postpartum. The women were randomized into the 3 intervention groups: iron-folic acid [IFA; Nutrition Products South Africa (Pty) Ltd], multiple micronutrients [MMN; Nutrition Products South Africa (Pty) Ltd], and small-quantity lipid-based nutrient supplements (LNS; Nutriset S.A.S.) (25). In brief, an independent researcher (not involved with the trial) created individual randomization slips in blocks of 9. The slips were then packed in sealed, numbered, and opaque randomization envelopes stored in numerical order. The enrolled women were asked to choose 1 of the top 6 envelopes in the stack, and the contents of each chosen envelope indicated her participant number and group allocation. Women in the IFA group (*n* = 290) received 60 mg of iron and 400 μg of folic acid in a capsule each day until delivery, followed by a placebo capsule (low dose calcium) until 6 mo postpartum. Women in the MMN group (*n* = 291) received 20 mg of iron, in addition to 400 μg of folic acid and 16 additional micronutrients each day until 6 mo postpartum. The IFA, placebo, and MMN capsules were identical in appearance. Women in the LNS group (*n* = 288) received a 20 g sachet of LNS each day until 6 mo postpartum. The LNS contained the same 18 micronutrients as the MMN capsule, with 4 additional minerals, protein and fat (from milk, peanuts, and soybean oil), and also provided 118 kcal of energy (25). No supplementation was given to children in the IFA and MMN groups, whereas children in the LNS group received another formulation of LNS from 6 to 18 mo. The 20 g daily LNS ration given to children was designed to meet micronutrient needs for healthy breastfeeding children (4, 24). All children were followed-up for 30 mo from birth. Supplements were delivered at the beginning of every 2-wk period. A statistician not involved in the study maintained the intervention code, which was not broken until all laboratory and statistical analyses were performed. Data on adherence of participants to the study intervention were recorded by counting the number of delivered and recovered capsules or sachets (25).

Dietary characteristics at 18 mo were assessed as reported previously (26). Briefly, dietary diversity and consumption of micronutrient‐rich food groups were assessed through a guided free recall of liquids and foods consumed by the child the previous day and a list‐based recall of the number of days each food group was consumed in the 7 d preceding the interview. Dietary diversity score was calculated based on report in 24-h recall (following WHO guidelines) as the sum of yes/no consumption of 7 defined food groups (grains, roots, and tubers; legumes and nuts; dairy products; flesh foods; eggs; vitamin A-rich fruits and vegetables; other fruits and vegetables), and consumption of animal‐source food groups yesterday was defined as having consumed ≥1 of organ meats, other meat/poultry, fish, eggs, or dairy.

The baseline sociodemographic information was collected by trained study personnel. The information gathered included age, household assets, and HIV status.

An asset index for each household was constructed based on ownership of a set of assets (radio, television, cell phone, bed, mattress, bed-net, and bicycle), lighting source, drinking water supply, sanitation facilities, and roofing materials. Household ownership of this set of assets was then combined into an index (with a mean of zero and SD of 1) with principal components analysis (27). Maternal HIV status was established with a whole-blood antibody rapid test (Alere Determine HIV-1/2, Alere Medical Co, Ltd and Uni-Gold HIV; Trinity Biotech plc) (25). Key details of the trial were recorded at the clinical trial registry at the National Institutes of Health (USA) (clinicaltrials.gov), under the registration number NCT01239693. The ethical clearance for the study was granted by the University of Malawi College of Medicine Research and Ethics Committee (COMREC) and the ethics committee at Tampere University Hospital District, Finland.

### Fecal sample collection

Fecal samples from participating children were collected by their mothers in their homes at ages 1, 6, 12, 18, and 30 mo, generally early in the morning, and at the end of every 2-wk supplementation delivery period. The mothers were informed of the upcoming sample collection visit and were provided with sample collection tubes the day before. On the day of sample collection, the mothers were asked if the child was passing normally formed feces (to exclude diarrhea). If diarrhea was suspected (>3 stools a day and markedly more liquid), the sample was not collected, and the visit was rescheduled for 2 wk later. Sample tubes were collected on the day of sample collection, usually in the morning. The tubes containing feces were sealed, labeled, and immediately stored in a Ziploc bag on a frozen ice pack in a cooler bag. The samples were subsequently transported to a satellite clinic and placed in a −20°C freezer for ≤2 d before being transported, frozen, to the central clinic in Mangochi for storage at −80°C. The samples were later packaged and shipped on dry ice to the USA for culture-independent analysis of community composition at Washington University, St. Louis, MO. Sample collection spanned all the 3 seasons of Malawi: warm-wet season, cool-dry-winter season, and hot-dry season.

### DNA purification and 16S rRNA sequencing

Details of DNA purification and generation and sequencing of amplicons produced from V4 of bacterial 16S rRNA genes have been described previously (20). Stool samples were homogenized by grinding in the presence of liquid nitrogen, followed by DNA extraction. DNA libraries were prepared by amplifying the V4 region (∼255 bp) of the 16S rRNA gene. Purified DNA libraries were then sequenced on the Illumina MiSeq platform. Sequence processing and picking of OTUs (at 97% sequence identity) were performed in QIIME (28). OTU data were filtered with a threshold of at least 0.1% of reads in ≥2 samples. OTUs are clusters of closely related sequences, at a defined similarity threshold (29). The V4 16S rRNA sequence data generated in this study are available through the European Nucleotide Archive (accession number PRJEB29433).

### Measurements of microbial maturity and diversity

Microbiota maturity was measured with a Random Forests machine-learning model (30, 31). This model, generated from analysis of fecal samples collected from members of a Malawian cohort from birth through the second year of life, predicts microbiota age based on the abundances of 25 age-discriminatory OTUs (30). Microbiota ages of study members predicted by this model were compared to the median microbiota age of chronologically age-matched children in the healthy reference group to generate microbiota-for-age *z* scores (MAZ).

Microbiota diversity and MAZ scores were calculated with OTU data that were rarefied to 5000 reads. Through use of the phyloseq package in R (32), we calculated the Shannon diversity index to measure the mean α diversity of OTUs in each sample. The Shannon index considers both richness and evenness of OTUs in each sample. A larger value indicates higher level of diversity (33). In addition, from the OTU data, species richness and Pielou's evenness function were calculated. Faith's phylogenetic diversity was calculated with the Picante R Package (34). We also measured β diversity with the Bray-Curtis metric (35) and tested whether samples were more similar within treatment groups than between groups. α Diversity, microbiota maturity (MAZ scores), and β diversity outcomes were assessed at 1, 6, 12, 18, and 30 mo.

### Statistical analysis

The primary analysis was based on the intention to treat principle. Our statistical analysis plan, posted at https://ilins.ucdavis.edu before beginning the analysis, specified that the analysis would first be performed with all 3 treatment groups, and if there was no difference between the IFA and MMN groups (in which no supplements were provided directly to the children), those 2 groups would be combined and the analyses would be repeated to test for differences between the LNS group and the other 2 groups combined. For the Shannon index, species richness, Pielou's evenness function, Faith's phylogenetic diversity and MAZ, outliers below the 2.5th percentile were truncated to the 2.5th percentile and outliers above the 97.5th percentile were truncated to the 97.5th percentile (36, 37). The primary analysis was completed with truncated outcome data, but a sensitivity analysis was performed with nontruncated data.

We first performed ANCOVA testing of differences between intervention groups, including child's age at measurement as a control covariate in every model. Additional adjustment covariates were included in adjusted models if they were related to the outcome when tested individually (*P* < 0.1). We tested the null hypothesis of no difference between the intervention groups with ANCOVA for all the outcomes.

The covariates considered were maternal HIV status at enrollment, season at time of stool sample collection, household assets *z* score at enrollment, and infant age at time of measurement. Potential effect modifiers were assessed with an interaction term in the ANCOVA. The potential effect modifiers were season at time of measurement, age at time of measurement, HIV status at baseline, and asset index at baseline. The 3 Malawian seasons include: warm-wet season (November–April); cool, dry winter season (May–August), and hot dry season (September–October) (38). Significant interactions (*P* < 0.05) were further examined with estimation of adjusted means within each category of the categorical effect modifier (HIV status and season) and at the 10th and 90th percentiles of the continuous effect modifier (age and household assets score), to understand the nature of the effect modification.

Differences in bacterial community composition between groups (β diversity) were assessed based on permutational multivariate analysis of variance (PERMANOVA) with distance matrices and permutation tests, and analysis of similarities (ANOSIM) (39, 40). To identify taxa that were significantly associated with a given nutritional intervention at each age, we conducted indicator species analyses (ISA). ISA identifies taxa that are statistically associated with an experimental or observational grouping with the indicator species value, the product of the probability that a given taxon is found in a sample belonging to a particular intervention group (*fidelity*), and the conditional probability that if the taxon is found, the sample in which it is found belongs to a particular intervention group (*specificity*) (41). Statistical significance of observed indicator species values was established by permutation tests, and *P* values were corrected for multiple comparisons with the Benjamini-Hochberg (BH) procedure at 10% false discovery rate.

## Results

### Study profile and follow-up outcome

Enrollment of pregnant women to the iLiNS-DYAD trial started on February 17, 2011 and was completed in August 2012. Out of the 869 mothers assigned to follow-up to 30 mo (Figure 1), 760 singleton live births were reported. The women who were not included experienced abortions/stillbirths (*n* = 20), dropped out of the study (*n* = 69), or gave birth to twins (*n* = 20). At 18 mo, 622 children completed anthropometric measurements and the rest (*n* = 138) were lost to follow-up. At 30 mo, 575 children completed anthropometric measurements. Stool samples were collected from 331 children at 1 mo, 515 at 6 mo, 630 at 12 mo, 622 at 18 mo, and 575 at 30 mo. We completed the 18-mo follow-up in April 2014 and the 30-mo follow-up in April 2015. Approximately 7% of visits were rescheduled across the sampling points between 1 and 18 mo of age. Loss to follow-up at all timepoints was similar across intervention groups. The mean adherence to the intervention was estimated at over 80% in all the groups (25).

### Study characteristics

At baseline, the distributions of maternal age at enrollment, household assets, and gestational age at birth were similar across intervention groups. The proportion of HIV positive mothers did not differ by intervention group, nor did the season at enrollment. The maternal age at enrollment and household assets *z* score at baseline for mothers excluded from this sub-study and for mothers included in the study were similar; however, gestational age at birth was slightly lower among those excluded (39.4 compared with 39.6 wk, *P* = 0.03) and HIV positive status was slightly less prevalent among those excluded (84% compared with 88%, *P* = 0.04). Indicators of dietary intake (at 18 mo) were not different by intervention (**Table 1**). Antibiotic use was rare, with reported use on ∼1% of observed days based on recall data collected during weekly home visits (24).

**TABLE 1:** Study characteristics of mothers included in the study by intervention group, characteristics of mothers excluded from the study, and dietary variables of children at 18 mo^1^

		Included, by intervention group						
Characteristic		IFA	MMN	LNS	*P * ^2^	Total included	Excluded	*P*
Participants, *n*		235	243	236		714	681	
Enrollment maternal age, y		24.5 (20.2, 29.9)	24.3 (20.6, 28.9)	24.4 (20.2, 29.3)	0.88	24.4 (20.3, 29.2)	24.0 (19.9, 28.7)	0.25
Gestational age at birth, wk		39.5 (38.5, 40.5)	39.6 (38.6, 40.5)	39.7 (38.8, 40.7)	0.46	39.6 (38.6, 40.6)	39.4 (38.0, 40.7)	0.03
Household assets *z* score		−0.39 (−0.74, 0.12)	−0.39 (−0.74, 0.12)	−0.39 (−0.74, 0.12)	0.43	−0.38 (−0.73, 0.12)	−0.37 (−0.74, 0.43)	0.87
HIV status	−ve	87	88	87	0.72	88	84	0.04
	+ve	13	12	13		12	16	
Season at sample collection^3^	Warm, wet	51	52	50	0.96	51		
	Cool, dry	27	27	30		28		
	Hot, dry	21	21	20		20		
Dietary variables at 18 mo	DDS^4^	3.52 ± 1.25	3.47 ± 1.32	3.67 ± 1.17	0.66	3.56 ± 1.25		
	Animal food^5^	72.9	66.2	71.7	0.28	70.2		
	Still breastfed	91.3	94.3	90.4	0.32	92.0		
	Breastfed ≥6 times previous day	61.1	72.4	67.0	0.06	66.8		

1 Values are medians (IQRs), means ± SDs, or percentages, unless otherwise indicated. DDS, dietary diversity score; IFA, iron and folic acid; LNS, lipid-based nutrient supplement; MMN, multiple micronutrients.

2 *P* value obtained from a nonparametric Kruskal–Wallis test (continuous variables) and chi-square test (categorical variables).

3 Season was modelled based on Malawian climate: warm, wet (November–April), cool, dry (May–August), and hot, dry (September–October).

4 Dietary data gathered from 24-h recall. Dietary diversity score calculated as the sum of yes/no consumption of 7 defined food groups (grains, roots, and tubers; legumes and nuts; dairy products; flesh foods; eggs; vitamin A-rich fruits and vegetables; other fruits and vegetables).

5 Consumption of animal‐source food groups yesterday defined as having consumed ≥1 of organ meats, other meat/poultry, fish, eggs, or dairy.

### Effect of intervention on microbiota characteristics

Our findings showed no significant differences in the microbiota diversity or MAZ scores between the IFA and MMN groups at any time point (**Supplemental Table 1**). Therefore, the IFA and MMN groups were combined into 1 control group (IFA + MMN) and the analyses repeated to test for differences between the LNS and control groups.

#### α Diversity

In the entire cohort of children, Shannon diversity index increased with age, with the mean ± SD values being 0.92 ± 0.50, 1.60 ± 0.59, 2.40 ± 0.65, 2.94 ± 0.59, and 3.53 ± 0.42 at 1, 6, 12, 18, and 30 mo, respectively. At 18 mo (at which point children had received LNS for 1 y), there was a difference of 0.10 in the mean α diversity values between the LNS and control groups (3.01 ± 0.57 compared with 2.91 ± 0.60, *P* = 0.032) (**Table 2**). Similarly, Pielou's evenness function was significantly higher in the LNS group than in the IFA + MMN group (*P* = 0.043) at 18 mo. There was a trend for species richness to be higher in the LNS group than in the IFA + MMN group (*P* = 0.08) at 18 mo. There was no significant difference in Faith's phylogenetic diversity at 18 mo between treatment groups (*P* = 0.19). At other ages [1 and 6 mo (without LNS supplementation); 12 mo (with LNS supplementation for half a year); and 30 mo (with children no longer supplemented for a year)], there were no significant differences between the groups in Shannon index, Faith's phylogenetic diversity, Pielou's evenness function, or species richness.

**TABLE 2:** Infant gut microbiota characteristics by intervention group at 1 mo, 6 mo, 12 mo, 18 mo, and 30 mo^1^

Microbiota characteristics	Time	IFA + MMN	LNS	IFA + MMN compared with LNS	*P * ^2^
Shannon diversity index	1 mo	0.88 ± 0.60 (225)^3^	0.99 ± 0.53 (106)	0.10 (−0.03, 0.22)	0.12
	6 mo	1.60 ± 0.66 (347)	1.59 ± 0.69 (168)	−0.01 (−0.11, 0.10)	0.86
	12 mo	2.39 ± 0.65 (419)	2.45 ± 0.72 (211)	0.01 (−0.10, 0.12)	0.90
	18 mo	2.91 ± 0.60 (414)	3.01 ± 0.57 (208)	0.11 (0.01, 0.21)	0.03
	30 mo	3.51 ± 0.47 (382)	3.58 ± 0.43 (193)	0.07 (−0.00, 0.14)	0.06
Pielou's evenness function	1 mo	0.25 ± 0.13 (225)	0.26 ± 0.13 (106)	0.01 (−0.01, 0.04)	0.32
	6 mo	0.39 ± 0.12 (347)	0.39 ± 0.12 (168)	0.00 (−0.03, 0.02)	0.76
	12 mo	0.52 ± 0.11 (413)	0.53 ± 0.12 (206)	0.00 (−0.02, 0.02)	0.83
	18 mo	0.60 ± 0.09 (414)	0.61 ± 0.08 (208)	0.02 (0.00, 0.03)	0.04
	30 mo	0.66 ± 0.06 (378)	0.67 ± 0.05 (192)	0.01 (−0.01, 0.02)	0.10
Faith's phylogenetic diversity	1 mo	6.45 ± 1.86 (225)	6.79 ± 1.93 (106)	0.34 (−0.10, 0.77)	0.13
	6 mo	11.15 ± 4.80 (347)	11.24 ± 5.17 (168)	0.00 (−0.82, 1.00)	0.84
	12 mo	16.33 ± 5.30 (404)	16.41 ± 5.58 (198)	0.09 (−0.79, 1.02)	0.85
	18 mo	21.30 ± 6.67 (414)	22.06 ± 7.05 (208)	0.76 (−0.38, 1.89)	0.19
	30 mo	30.75 ± 7.85 (378)	31.28 ± 6.90 (192)	0.67 (−0.78,1.84)	0.43
Species richness	1 mo	32.5 ± 10.9 (225)	34.8 ± 12.6(106)	2.22 (−0.44, 4.87)	0.10
	6 mo	60.3 ± 25.3 (347)	59.4 ± 24.0 (168)	−0.84 (−5.43, 3.76)	0.72
	12 mo	97.8 ± 39.3 (414)	98.0 ± 40.4 (206)	0.18 (−6.47, 6.83)	0.96
	18 mo	134.9 ± 51.6 (414)	142.9 ± 55.4 (208)	7.97 (−0.85, 16.79)	0.08
	30 mo	214.4 ± 60.9 (378)	219.8 ± 54.7 (192)	5.39 (−4.86, 15.64)	0.30
MAZ^4^	1 mo	NA	NA	NA	NA
	6 mo	0.63 ± 2.39 (347)	0.40 ± 2.47 (168)	−0.23 (−0.66, 0.20)	0.30
	12 mo	−0.33 ± 2.52 (419)	−0.27 ± 2.80 (211)	0.06 (−0.36, 0.49)	0.77
	18 mo	−1.37 ± 1.70 (414)	−1.27 ± 1.81 (208)	0.10 (−0.19, 0.39)	0.49
	30 mo	−3.70 ± 2.37 (382)	−3.50 ± 2.43 (193)	0.19 (−0.21, 0.62)	0.35

1 Values are means ± SDs (*n*) or mean differences (95% CI). IFA + MMN, iron and folic acid + multiple micronutrients; LNS, lipid-based nutrient supplement; MAZ, microbiota-for-age *z* score; NA, not applicable.

2 *P* values obtained from ANCOVA.

3 Number of infants at each sampling time point stratified by intervention group.

4 No data for the calculation of MAZ were available at 1 mo.

**FIGURE 2:**
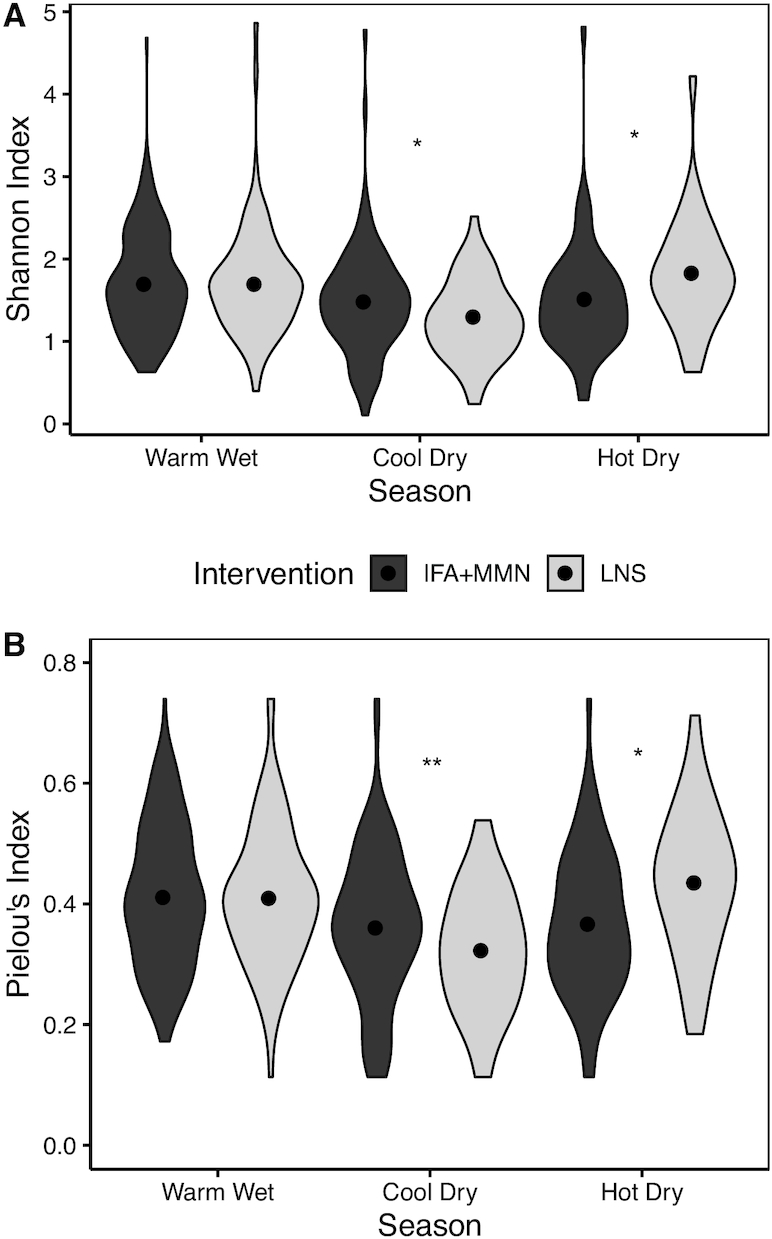
Shannon index (A) and Pielou's index (B) at 6 mo as a function of season at the time of sample collection by intervention group. Sample sizes for IFA + MMN compared with LNS groups were as follows: warm-wet season (246 compared with 118), cool-dry season (129 compared with 71), and hot-dry season (101 compared with 47). *IFA + MMN and LNS groups differ, *P* < 0.05. **IFA + MMN and LNS groups differ, *P* < 0.01. IFA, iron and folic acid; LNS, lipid-based nutrient supplement; MMN, multiple micronutrients.

**FIGURE 3:**
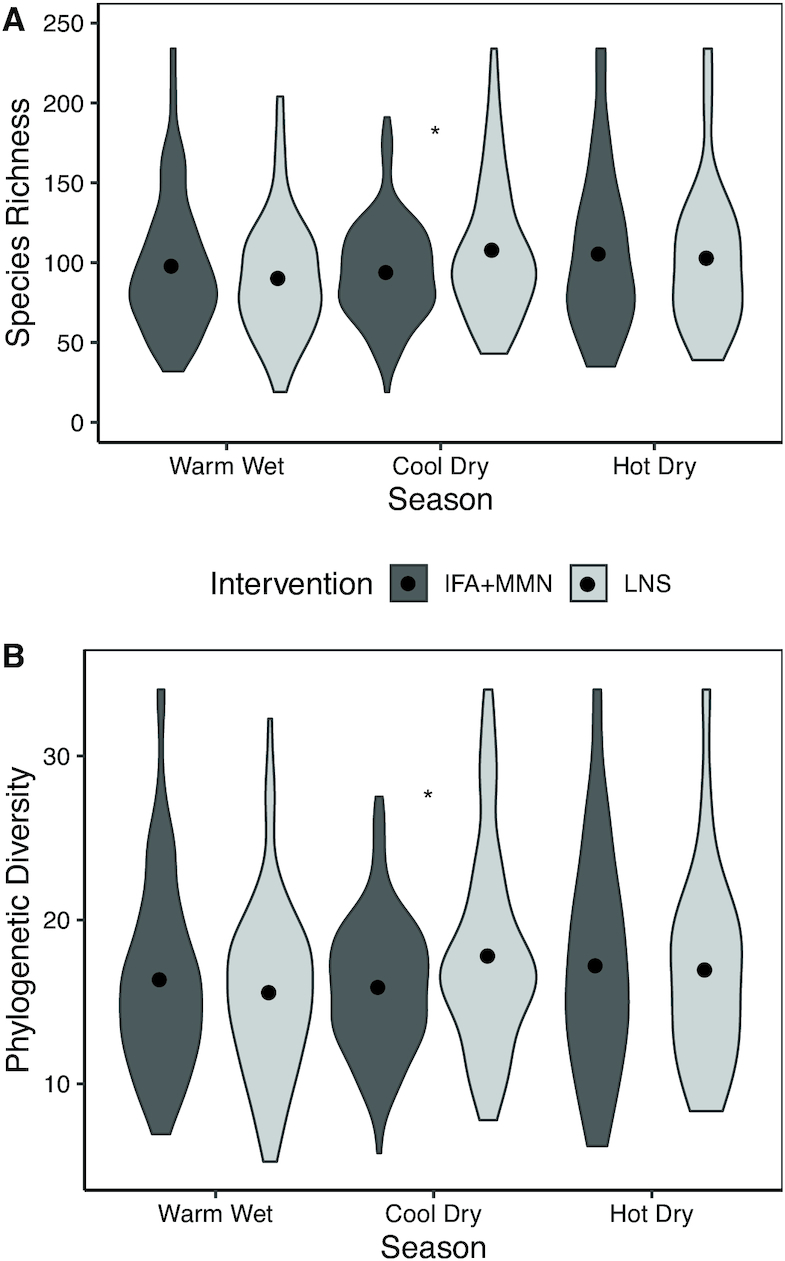
Species richness (A) and phylogenetic diversity (B) at 12 mo as a function of season at the time of sample collection by intervention group. Sample sizes for IFA + MMN compared with LNS groups were as follows: warm-wet season (246 compared with 118), cool-dry season (129 compared with 71), and hot-dry season (101 compared with 47). *IFA + MMN and LNS groups differ, *P* < 0.05. IFA, iron and folic acid; LNS, lipid-based nutrient supplement; MMN, multiple micronutrients.

Regarding potential effect modifiers, season at the time of sample collection modified the association between dietary intervention and microbial diversity at ages 6 and 12 mo. At 6 mo, Shannon diversity and Pielou's evenness were significantly higher in the LNS group compared with the IFA + MMN group (*P* = 0.012, 0.042, respectively) in the hot-dry season, but they were lower in the LNS group in the cool, dry winter (*P* < 0.05, < 0.01, respectively), and there was no significant group difference during the warm-wet season (*P* = 0.77, 0.85, respectively) (**Figure 2**). At 12 mo, species richness and phylogenetic diversity were higher in the LNS group (compared with the IFA + MMN group, *P* = 0.019 and *P* = 0.012, respectively) in the cool, dry season but not in the other 2 seasons (**Figure 3**).

**FIGURE 4:**
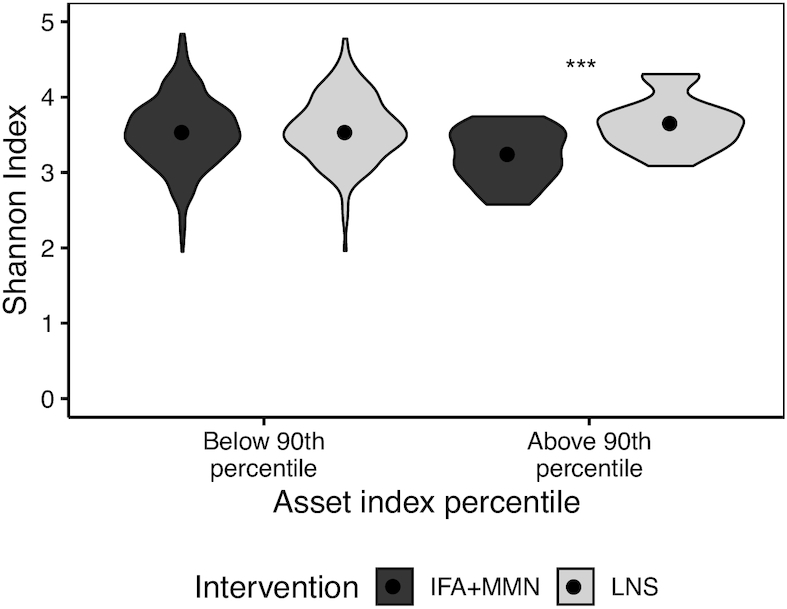
Shannon index at 30 mo as a function of baseline asset index percentile by intervention group. Sample sizes for IFA + MMN compared with LNS groups were as follows: below 90th percentile (351 compared with 170) and above 90th percentile (26 compared with 20). ***IFA + MMN and LNS groups differ, *P* < 0.001. IFA, iron and folic acid; LNS, lipid-based nutrient supplement; MMN, multiple micronutrients.

Household asset *z* score modified the association between the intervention group and diversity at 30 mo: among children in households with higher asset scores, Shannon diversity index was significantly higher in those who received LNS compared with IFA + MMN (*P* < 0.001), whereas no differences were observed among children in households with lower asset scores (**Figure 4**). A similar pattern was observed for Faith's phylogenetic diversity, Pielou's evenness index, and species richness at 30 mo.

#### β Diversity

Based on PERMANOVA and ANOSIM, we did not detect any significant effects of LNS intervention on the bacterial composition of the microbiota in any age group (*P* > 0.05; **Supplemental Figure 1**). However, significant effects of age on bacterial community composition were detected by both PERMANOVA (*R^2^* = 0.32, pseudo-F = 314, *P* < 0.001) and ANOSIM (*R* = 0.4514, *P* < 0.001) (**Supplemental Figure 2**).

#### Microbiota maturity

In the entire cohort of children, MAZ declined with age, with the mean ± SD MAZ scores being 0.64 ± 2.41, −0.28 ± 2.57, −1.32 ± 1.72, and −3.69 ± 2.33 at 6, 12, 18, and 30 mo, respectively. There were no significant differences in MAZ between the intervention groups at any age when this metric was calculated (Table 2), and no significant effect modifiers. The MAZ metric was not calculated for samples at 1 mo because of the low sample size at this age in the healthy reference control cohort that was used to generate the Random Forests-derived model.

#### Taxa distribution profile

The taxonomic profile (at genus level) and the corresponding relative abundances were similar by intervention group at each age (**Supplemental Figure 3**). However, the number of genera detected increased with age, accompanied by an increase or decrease in the abundances of specific genera. The most abundant genus at 1 mo (*Bifidobacterium*, predominantly *Bifidobacterium longum* species) gradually decreased with age to become 1 of the least abundant species at 30 mo. At the same time, the abundance of *Prevotella* (predominantly *Prevotella copri* species) increased with age, emerging as 1 of the most abundant species at 30 mo. Although the abundance of *Streptococcus* remained relatively stable with age, the abundance of *Faecalibacterium* was seen to increase from 6 up to 30 mo.

#### Indicator species analysis

**FIGURE 5:**
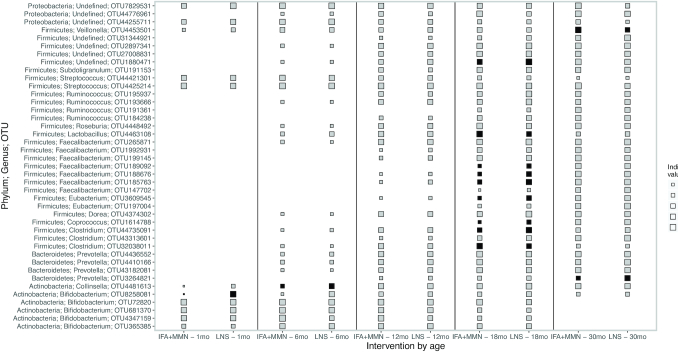
Indicator species analysis at genus level by intervention at 1 mo, 6 mo, 12 mo, 18 mo, and 30 mo. The size of each square is proportional to the size of the indicator species value metric. Sample sizes at each time point for the IFA + MMN compared with LNS groups were as follows: 1 mo (225 compared with 106), 6 mo (347 compared with 168), 12 mo (419 compared with 211), 18 mo (414 compared with 208), and 30 mo (382 compared with 193). () IFA + MMN and LNS groups differ, *P* < 0.05 (before the Benjamini-Hochberg correction). IFA, iron and folic acid; LNS, lipid-based nutrient supplement; MMN, multiple micronutrients.

To identify taxa whose abundances differed between the 2 intervention arms, we conducted ISA at OTU level at each age (**Figure 5**). At 1 mo there was only 1 indicator OTU (OTU_8,258,081), which belonged to the *Bifidobacterium* genus. At 6 mo we detected 1 indicator OTU (OTU_4,481,613), which belonged to *Collinsella aerofaciens* species. These 2 OTUs belonged to phylum Actinobacteria and were both significantly positively associated with LNS intervention. Whereas no indicator OTUs were detected at 12 mo, we detected 9 indicator OTUs 18 mo and 2 at 30 mo. All the 9 indicator OTUs detected at 18 mo belonged to the phylum Firmicutes; for 7 of these OTUs, prevalence was higher in the LNS group (these OTUs included genera *Clostridium, Eubacterium, Coprococcus*, and *Faecalibacterium prausnitzii* species), while for 2 OTUs (representing *Clostridium* and *Lactobacillus ruminis*), prevalence was higher in the control group. Of the 2 indicator OTUs at 30 mo, 1 was significantly positively associated with the LNS intervention (OTU_3,264,821, a *Prevotella* genus belonging to Bacteroidetes phyla) and the other with the control group (OTU_4,453,501, a *Veillonella atypica* species belonging to Firmicutes phyla). However, following BH correction at 10% false discovery rate, none of the observed associations remained significant.

## Discussion

We tested the hypothesis that supplementation with LNS to mothers during pregnancy and the first 6 mo postpartum and to infants between the ages of 6 and 18 mo would lead to a more diverse or mature gut microbiota between the ages of 1 mo and 30 mo, compared to supplementation with MMN during pregnancy and the first 6 mo postpartum only, or IFA during pregnancy only. α Diversity (based on the Shannon diversity index and Pielou's evenness) was higher in the LNS group at age 18 mo but no group differences were observed at ages 1, 6, 12, or 30 mo. There was no significant difference between intervention groups in species richness, Faith's phylogenetic diversity, or microbiota maturity as determined by MAZ score at any age. β Diversity (based on Bray-Curtis dissimilarity) was not affected by the intervention, although a distinct clustering was observed by age group. Through use of ISA, we established associations of certain taxa at OTU level with intervention group (IFA + MMN or LNS) at 6, 12, 18, and 30 mo; however, these did not remain significant following BH correction.

Strengths of this study include random allocation of study participants to intervention groups, blinding of the outcome assessors to the intervention, training of research personnel in good clinical practices, and a high level of standardization and quality assurance during data collection. One of the limitations was that there were temporary interruptions of the LNS distribution during the trial, partly because of temporary migration out of the study area and inability of the research personnel to locate the participant(s) during active farming periods. However, the interruptions were not major, and the proportion of missing participants did not differ between groups. A potential source of bias is that there were statistically significant differences in 2 baseline characteristics (gestational age at birth, HIV status) between included and excluded participants. However, the difference of 0.2 wk in gestational age was small and these characteristics were not related to the main microbiota outcomes (α diversity and MAZ), so our findings should be generalizable to the study population. Sample handling may affect the profile of fecal microbiota, especially if immediate storage at −80°C is not feasible (42). Time between sample collection and storage would have differed by child-specific logistical constraints and may affect results; however, data collector blinding prevents these differences from being systematic by intervention group.

The significant difference in α diversity measured by Shannon index (LNS > control group) and by Pielou's index (LNS > control group) at 18 mo but not at 1 or 6 mo would suggest that the maternal component of the intervention did not have a detectable influence on the child's microbiota. By 18 mo, the children in the LNS group had received supplements for 1 y, while at 30 mo they had been without supplementation for 1 y. Thus, it is not surprising that the overall difference in α diversity between supplementation groups would be seen at 18 mo but not at 30 mo, although it is also possible that the difference at 18 mo was a random finding. The fact that we saw no difference in α diversity at 12 mo suggests that 6 mo of LNS was insufficient to change this outcome. By 30 mo, other factors may have attenuated the difference that was evident at 18 mo. Such factors may include the cessation of breastfeeding and increased consumption of solid foods, which have been linked to changes in microbial community composition between 9 and 18 mo (43).

Season appeared to modify the association of intervention with certain outcomes at 6 and 12 mo. At 6 mo, there was higher α diversity (Shannon and Pielou's indices) in the LNS group than in the IFA + MMN group during the hot-dry season, whereas in the cool-dry season, α diversity was higher in the IFA + MMN group than in the LNS group. The potential mechanism by which maternal LNS consumption might influence the child's microbiome diversity in different ways in the hot-dry compared with cool-dry seasons is unclear, given that no significant impact of the intervention on bioactive breast milk proteins or oligosaccharides at 6 mo was reported (44). At 12 mo, the pattern was different from that observed at 6 mo: species richness and phylogenetic diversity were higher in the LNS group (compared to the IFA + MMN group) during the cool-dry season, with no differences observed in the hot-dry or warm-wet seasons. Thus, the interactions between season and the LNS intervention are difficult to explain and could be a result of chance.

Household wealth, as measured by the assets score, appeared to modify whether the intervention was associated with microbial diversity at 30 mo: among children in households with greater assets, all 4 indices of diversity were higher in the LNS group compared to the IFA + MMN group, whereas no differences were observed among children in poorer households. There is a possibility that weaning and dietary intake contribute to the effect modification by socioeconomic status observed at 30 mo. We do not have dietary data at 30 mo, but at 18 mo, children in households with assets scores above the median were less likely to still be breastfed (85% compared with 94%) and had better quality diets in terms of dietary diversity and consumption of animal-source foods (data not shown). Previous studies have shown that higher bacterial diversity is associated with lower socioeconomic status among pre-adolescents in Malaysia (45) and children aged <5 y in Kenya (46). The higher microbial diversity among children of poorer households may mask the effect of LNS intervention in our setting.

The LNS intervention was associated with the abundance of certain OTUs at 1, 6, 18, and 30 mo but the differences were not statistically significant following correction for multiple hypothesis testing. This is generally consistent with findings from an earlier study in Malawi (20), which showed that supplementation of children with LNS, or corn-soya blend for 12 mo, did not affect microbiota composition at 6 or 18 mo of age. Another study in which Malawian children aged 6 mo received LNS for 6 mo also reported no effect of LNS intervention on the prevalence or counts of *Bifidobacterium* in children aged 12 mo (21). In all studies conducted in Malawi (including this study), breastfeeding was universal and typically of long duration, which may reduce the likelihood of detecting effects of a nutrition intervention.

OTUs representing genera such as *Shigella, Escherichia*, or *Salmonella*, which may be pathogenic, were detected at low abundance levels in our study. These OTUs did not show any association with either IFA + MMN or LNS at any age even before correction for multiple hypothesis testing, which is consistent with previous findings from the same setting (20). In Kenya (19), supplementation with iron-fortified micronutrient powder [MNP, containing iron as sodium iron EDTA (NaFeEDTA, ± 2.5 mgFeMNP)] was associated with an increase in *Escherichia coli* and *Shigella*. Our trial used LNS rather than MNP, but it is also possible that variations in the formulation and amount of iron provided accounts for the different findings, as the Malawian children received less iron per dose (6 mg) than that provided in the Kenyan study (12.5 mg) (19).

The increase in α diversity with age is consistent with findings from a longitudinal study of Norwegian children (47). The differences in composition by age category are also consistent with other results demonstrating the development of a more stable microbial community over time (47). By contrast, the MAZ score decreased with age, suggesting worsening relative microbiota maturity in this cohort of Malawian children, which parallels the worsening of height-for-age *z* scores (48).

We conclude that providing LNS to mothers during pregnancy and 6 mo postpartum and to their infants from 6 to 18 mo promotes infant gut microbiota diversity at 18 mo, and that a persistent effect of the intervention 12 mo postsupplementation may be evident depending on the participants’ socioeconomic status. The findings did not support the hypothesis that LNS supplementation will promote gut microbiota maturity in Malawian infants. Changes in microbial diversity may lead to changes in the functional capacity of the microbial community (49), but whether greater diversity would affect biological processes mediated by these microorganisms remains largely unknown. Recently, we showed that microbiota diversity and maturity were related to growth in weight; however, their impact on inflammation was inconclusive (50), necessitating the need for further investigation.

## Supplementary Material

nxz298_Supplemental_File

## Notes

Supported by the Bill & Melinda Gates Foundation grant number OPP49817.

Author disclosures: The authors report no conflicts of interest.

The data described in the manuscript, the code book, and the analytic code will be made available to the editors upon request.

Supplemental Table 1 and Supplemental Figures 1–3 are available from the “Supplementary data” link in the online posting of the article and from the same link in the online table of contents at https://academic.oup.com/jn.

Abbreviations used: BH, Benjamini-Hochberg procedure; IFA, iron and folic acid; ISA, indicator species analysis; LNS, lipid-based nutrient supplement; MAZ, microbiota-for-age *z* score; MMN, multiple micronutrients; MNP, micronutrient powder; OTU, operational taxonomic unit; SQ-LNS, small-quantity lipid-based nutrient supplement.
