# Supplementary material for: Provision of Lipid-Based Nutrient Supplements to Mothers During Pregnancy and 6 Months Postpartum and to Their Infants from 6 to 18 Months Promotes Infant Gut Microbiota Diversity at 18 Months of Age but Not Microbiota Maturation in a Rural Malawian Setting: Secondary Outcomes of a Randomized Trial
Source: J Nutr. 2020 Jan 7;150(4):918–28. doi: 10.1093/jn/nxz298 (PMC7138685; doi:10.1093/jn/nxz298)
Supplement: nxz298_Supplemental_File [file nxz298_supplemental_file.docx]

**Supplemental Table 1** Infant gut microbiota characteristics by intervention group at 1 mo, 6 mo, 12 mo, 18 mo, and 30 mo^1^

|  |  |  |  |  |  |  | LNS vs IFA | |  | MMN vs IFA | |  | LNS vs MMN | |
| --- | --- | --- | --- | --- | --- | --- | --- | --- | --- | --- | --- | --- | --- | --- |
| Microbiota characteristics | Time | IFA | MMN [n] | LNS | *P*^2^ |  |  | *P*^2^ |  |  | *P*^2^ |  |  | *P*^2^ |
| Shannon  Diversity Index | 1 mo | 0.85±0.05 [103]^3^ | 0.90±0.05 [122] | 0.95±0.05 [106] | 0.39 |  | 0.1  (-0.07, 0.26) | 0.35 |  | 0.04  (-0.11, 0.20) | 0.79 |  | -0.05  (-0.21, 0.11) | 0.72 |
|  | 6 mo | 1.56±0.04  [170] | 1.63±0.04 [177] | 1.58±0.04 [168] | 0.53 |  | 0.01  (-0.13, 0.16) | 0.98 |  | 0.07  (-0.08, 0.21) | 0.54 |  | 0.05  (-0.09, 0.20) | 0.66 |
|  | 12 mo | 2.43±0.04 [209] | 2.35±0.04  [210] | 2.41±0.04 [211] | 0.45 |  | -0.01  (-0.16, 0.13) | 0.97 |  | -0.08  (-0.22, 0.07) | 0.46 |  | -0.06  (-0.21, 0.09) | 0.60 |
|  | 18 mo | 2.88±0.04  [201] | 2.94±0.04 [213] | 3.01±0.04 [208] | 0.08 |  | 0.13  (-0.01, 0.27) | 0.07 |  | 0.05  (-0.08, 0.19) | 0.63 |  | -0.07  (-0.21, 0.06) | 0.39 |
|  | 30 mo | 3.51±0.03 [200] | 3.52±0.03 [182] | 3.58±0.03 [193] | 0.18 |  | 0.07  (-0.03, 0.17) | 0.22 |  | 0  (-0.10, 0.10) | 1.00 |  | -0.07  (-0.17, 0.03) | 0.26 |
| MAZ^4^ | 1 mo | na | na | na | na |  | na | na |  | na | na |  | na | na |
|  | 6 mo | 0.52±0.18  [170] | 0.71±0.17 [177] | 0.34±0.18 [168] | 0.33 |  | -0.18  (-0.77, 0.41) | 0.76 |  | 0.19  (-0.39, 0.78) | 0.72 |  | 0.37  (-0.21, 0.96) | 0.30 |
|  | 12 mo | -0.27±0.18 [209] | -0.40±0.18 [210] | -0.22±0.18 [211] | 0.76 |  | 0.04  (-0.54, 0.63) | 0.98 |  | -0.13  (-0.72, 0.45) | 0.85 |  | -0.18  (-0.76, 0.41) | 0.76 |
|  | 18 mo | -1.41±0.12 [201] | -1.31±0.12 [213] | -1.26±0.12 [208] | 0.67 |  | 0.15  (-0.25, 0.55) | 0.65 |  | 0.10  (-0.30, 0.50) | 0.82 |  | -0.05  (-0.44, 0.35) | 0.96 |
|  | 30 mo | -3.71±0.16 [200] | -3.71±0.17 [182] | -3.50±0.17 [193] | 0.59 |  | 0.21  (-0.34, 0.77) | 0.64 |  | 0  (-0.56, 0.56) | 1.00 |  | -0.21  (-0.78, 0.35) | 0.65 |
| Faith’s Phylogenetic Diversity | 1 mo | 6.54±1.87 [103] | 6.42±1.88 [122] | 6.54±1.87 [106] | 0.31 |  | 0.31  (-0.31, 0.92) | 0.47 |  | -0.06 (-0.65, 0.54) | 0.97 |  | -0.36  (-0.95, 0.23) | 0.31 |
|  | 6 mo | 10.68±0.38  [170] | 11.60±0.37 [177] | 11.24±0.38 [168] | 0.20 |  | 0.56  (-0.70, 1.82) | 0.55 |  | 0.91  (-0.70, 1.82) | 0.78 |  | 0.35  (-0.89, 1.60) | 0.95 |
|  | 12 mo | 16.56±0.38 [206] | 16.11±0.37 [207] | 16.45±0.38 [206] | 0.68 |  | -0.11  (-1.36, 1.14) | 0.98 |  | -0.34  (-1.58, 0.91) | 0.80 |  | -0.45  (-1.70, 0.80) | 0.68 |
|  | 18 mo | 20.77±0.48 [201] | 21.81±0.47 [213] | 22.06±0.47 [208] | 0.13 |  | 1.30  (-0.28, 2.87) | 0.13 |  | 1.04  (-0.53, 2.61) | 0.26 |  | 0.25  (-1.31, 1.81) | 0.92 |
|  | 30 mo | 31.12±0.53 [199] | 30.34±0.56 [179] | 31.28±0.54 [192] | 0.44 |  | 0.16  (-1.63, 1.95) | 0.98 |  | -0.94  (-2.78, 0.90) | 0.45 |  | 0.78  (-1.04, 2.61) | 0.57 |
| Pielou’s Evenness Function | 1 mo | 0.24±0.10  [103] | 0.25±0.10 [122] | 0.26±0.10 [106] | 0.50 |  | 0.02  (-0.02, 0.06) | 0.47 |  | 0.02  (-0.03, 0.05) | 0.81 |  | -0.01  (-0.05, 0.03) | 0.82 |
|  | 6 mo | 0.38±0.01  [170] | 0.40±0.01 [177] | 0.39±0.01 [168] | 0.64 |  | 0.00  (-0.03, 0.03) | 0.98 |  | 0.01  (-0.02, 0.04) | 0.64 |  | 0.01  (-0.03, 0.03) | 0.76 |
|  | 12 mo | 0.53±0.01 [206] | 0.52±0.01 [207] | -0.53±0.01 [206] | 0.38 |  | -0.01  (-0.03, 0.02) | 0.87 |  | 0.01  (-0.01, 0.04) | 0.35 |  | 0.01  (-0.02, 0.03) | 0.66 |
|  | 18 mo | 0.59±0.01 [201] | 0.60±0.01 [213] | 0.61±0.01 [208] | 0.12 |  | 0.02  (-0.00, 0.04) | 0.13 |  | 0.00  (-0.02, 0.02) | 0.90 |  | 0.01  (-0.01, 0.03) | 0.27 |
|  | 30 mo | 0.66±0.00 [199] | 0.66±0.00 [179] | 0.67±0.00 [192] | 0.24 |  | 0.01  (-0.00, 0.02) | 0.23 |  | -0.01  (-0.02, 0.01) | 0.44 |  | 0.00  (-0.02, 0.01) | 0.93 |
| Species Richness | 1 mo | 31.9±1.17 [103] | 32.7±1.09 [122] | 34.8±1.11 [106] | 0.21 |  | 2.80  (-0.94, 6.54) | 0.18 |  | 1.08  (-2.53, 4.70) | 0.76 |  | -1.72  (-5.31, 1.87) | 0.50 |
|  | 6 mo | 57.96±1.91  [170] | 62.51±1.87 [177] | 59.44±1.92 [168] | 0.22 |  | 1.48  (-4.87, 7.83) | 0.85 |  | 4.55  (-1.72, 10.82) | 0.20 |  | 3.07  (-3.22, 9.35) | 0.85 |
|  | 12 mo | 99.42±2.77 [206] | 96.13±2.76 [207] | 97.95±2.77 [206] | 0.67 |  | 1.47  (-7.72, 10.66) | 0.93 |  | -3.3  (-12.48, 2.88) | 0.89 |  | 1.83  (-7.35, 11.00) | 0.76 |
|  | 18 mo | 131.24±3.72 [201] | 138.41±3.62 [213] | 142.90±3.66 [208] | 0.08 |  | 11.66  (-0.61, 23.93) | 0.07 |  | 7.17  (-5.03, 19.37) | 0.35 |  | 4.49  (-7.60, 16.58) | 0.66 |
|  | 30 mo | 216.8±4.17 [199] | 211.7±4.4 [179] | 219.8±4.25 [192] | 0.41 |  | 2.95  (-11.10, 16.95) | 0.87 |  | -5.14  (-19.40, 9.11) | 0.67 |  | 8.09  (-6.28, 22.47) | 0.38 |

^1^Values are means ± SDs (*n*) or mean differences (95% CI). IFA, iron and folic acid; LNS, lipid-based nutrient supplement; MAZ, microbiota for age *Z*-score; MMN, multiple micronutrients; na, not applicable (no data for the calculation of MAZ was available at 1 mo).

^2^*P* values obtained from ANCOVA).

^3^Number of infants at each sampling time point stratified by intervention group.

^4^ No data for the calculation of MAZ was available at 1 mo.

**Supplemental Figure 1** Principal coordinate analysis (PCoA) by intervention group at 1 mo (A), 6 mo (B), 12 mo (C), 18 mo (D) and 30 mo (E). Each shape represents a sample and each color corresponds to samples belonging to each of the two intervention groups. Samples sizes at each time point for IFA+MMN vs LNS groups were as follows: 1 mo (225 vs 106), 6 mo (347 vs 168), 12 mo (419 vs 211), 18 mo (414 vs 208) and 30 mo (382 vs 193). IFA+MMN, iron and folic acid + multiple micronutrients; LNS, lipid-based nutrient supplement.

**Supplemental Figure 2** Principal coordinate analysis (PCoA) by age. Each shape represents a sample and each color corresponds to samples belonging to a particular age category.

Samples sizes at each time point were as follows: 1 mo = 331, 6 mo = 515, 12 mo = 630, 18 mo = 622 and 30 mo = 575. Microbiota differences by age category are significant according to PERMANOVA (*R^2^*=0.320, pseudo-F=314, *P*<0.001) and ANOSIM (*R*=0.4514, *P*<0.001).

**Supplemental Figure 3** The relative abundance of taxa detected at genus level as a function of intervention group at 1 mo, 6 mo, 12 mo, 18 mo, and 30 mo. The color coding in each bar graph correspond to different types of genera present at each time point. Samples sizes at each time point for IFA+MMN vs LNS groups were as follows: 1 mo (225 vs 106), 6 mo (347 vs 168), 12 mo (419 vs 211), 18 mo (414 vs 208) and 30 mo (382 vs 193). IFA+MMN, iron and folic acid + multiple micronutrients; LNS, lipid-based nutrient supplement.
